# Validation of a Musculoskeletal Digital Assessment Routing Tool: Protocol for a Pilot Randomized Crossover Noninferiority Trial

**DOI:** 10.2196/31541

**Published:** 2021-12-13

**Authors:** Cabella Lowe, Harry Hanuman Sing, William Marsh, Dylan Morrissey

**Affiliations:** 1 Centre for Sports & Exercise Medicine William Harvey Research Institute Queen Mary University of London London United Kingdom; 2 Risk and Information Systems Research Group School of Electronic Engineering and Computer Science Queen Mary University of London London United Kingdom; 3 Department of Physiotherapy Barts Health NHS Trust London United Kingdom

**Keywords:** mHealth, mobile health, eHealth, digital health, digital technology, musculoskeletal, triage, physiotherapy triage, validation, mobile phone

## Abstract

**Background:**

Musculoskeletal conditions account for 16% of global disability, resulting in a negative effect on millions of patients and an increasing demand for health care use. Digital technologies to improve health care outcomes and efficiency are considered a priority; however, innovations are rarely tested with sufficient rigor in clinical trials, which is the gold standard for clinical proof of safety and efficacy. We have developed a new musculoskeletal digital assessment routing tool (DART) that allows users to self-assess and be directed to the right care. DART requires validation in a real-world setting before implementation.

**Objective:**

This pilot study aims to assess the feasibility of a future trial by exploring the key aspects of trial methodology, assessing the procedures, and collecting exploratory data to inform the design of a definitive randomized crossover noninferiority trial to assess DART safety and effectiveness.

**Methods:**

We will collect data from 76 adults with a musculoskeletal condition presenting to general practitioners within a National Health Service (NHS) in England. Participants will complete both a DART assessment and a physiotherapist-led triage, with the order determined by randomization. The primary analysis will involve an absolute agreement intraclass correlation (A,1) estimate with 95% CI between DART and the clinician for assessment outcomes signposting to condition management pathways. Data will be collected to allow the analysis of participant recruitment and retention, randomization, allocation concealment, blinding, data collection process, and bias. In addition, the impact of trial burden and potential barriers to intervention delivery will be considered. The DART user satisfaction will be measured using the system usability scale.

**Results:**

A UK NHS ethics submission was done during June 2021 and is pending approval; recruitment will commence in early 2022, with data collection anticipated to last for 3 months. The results will be reported in a follow-up paper in 2022.

**Conclusions:**

This study will inform the design of a randomized controlled crossover noninferiority study that will provide evidence concerning mobile health DART system clinical signposting in an NHS setting before real-world implementation. Success should produce evidence of a safe, effective system with good usability, potentially facilitating quicker and easier patient access to appropriate care while reducing the burden on primary and secondary care musculoskeletal services. This rigorous approach to mobile health system testing could be used as a guide for other developers of similar applications.

**Trial Registration:**

ClinicalTrials.gov NCT04904029; http://clinicaltrials.gov/ct2/show/NCT04904029

**International Registered Report Identifier (IRRID):**

PRR1-10.2196/31541

## Introduction

### Background

Musculoskeletal disorders (MSDs) are the leading contributors to years lived with disability worldwide and have shown an increase in disease burden over the past decade [1-3]. Musculoskeletal conditions can affect as many as 1 in 4 adults and are set to continue rising, being associated with decreased life expectancy and reduced activity [4,5]. MSDs are prevalent throughout the life span and associated with early work retirement and reduced ability to participate socially [5]. In developed countries, they present the most significant proportion of lost productivity in the workplace, leading to a significant impact on the gross domestic product and health care costs [6,7].

In the United Kingdom, this poses a financial and societal challenge, costing >£4.76 billion (US $6.35 billion) of the UK National Health Service (NHS) resources and using up to 30% of primary care physician visits annually [8,9]. A freedom of information request has revealed that the average waiting time for NHS musculoskeletal outpatient physiotherapy services exceeded 6 weeks in the year to April 2019, with some patients waiting 4 months for routine appointments [10]. Longer waiting times can result in delays to physiotherapy services, with potentially detrimental effects on pain, disability, and quality of life [11,12]. The increasing proportion of burdens on public health services because of MSDs has highlighted the need for a targeted policy response [3,13].

Access to the *right person, right place, first time* is considered a key factor in improving musculoskeletal condition outcomes and reducing unwarranted variation in clinical pathways, such as unnecessary secondary care consultations and investigations [14]. Musculoskeletal triage as a single point of access is effective across various outcome measures, including user satisfaction, diagnostic agreement, appropriateness of referral, and reduction in patient waiting times [15]. Importantly, triage has also shown a reduction in costs across the musculoskeletal pathway, which is particularly crucial in overburdened health care systems, where triage can be performed effectively via several methods and by a range of clinicians [16-18].

Remote triage services such as telephone and video consultations or web-based or digital applications have the potential to reduce waiting times and musculoskeletal caseload [15,19]. Direct access to these services with initial assessments by physiotherapists may be a viable, cost-effective solution for managing the growing burden of MSD demand and workloads [19-22], with recent advances being made in digital primary care triage applications [23,24]. Some research has suggested that physiotherapy-led telephone triage is shown to be clinically as effective as usual care [21,25] and broadly acceptable to patients with MSDs seeking early physiotherapy advice [26]. However, barriers include the time required to reach a triage outcome, limited patient and professional trust, and interoperability problems. It should also be noted that comparisons between studies are hampered by variations in outcome measures and lack of randomization and statistical power, making any generalizations across health care settings problematic.

Mobile health (mHealth) technology has been proposed as a cost-effective solution for improving health care delivery [27,28]; however, this requires robustly tested and validated web-based triage platforms to signpost patients with MSDs to an appropriate level of care [19,29]. Standards and guidance for safe and effective implementation of mHealth apps have been published by several national and international organizations, all specifying a requirement for evidence of clinical safety and effectiveness [30-38]. A UK evidence standards framework specifically requires as the best practice standard a high-quality randomized controlled study or studies done in settings relevant to the UK health and social care system, which compare the digital health technology with a relevant comparator and demonstrate consistent benefits, including clinical outcomes in the target population [30].

To date, there is limited evidence regarding the use of web-based or digital triage platforms for MSDs specifically, and most investigations have focused on the performance of generic symptom checkers, covering a wide range of clinical presentations. However, the evidence from these studies concerning clinical- and cost-effectiveness, signposting to appropriate services, patient compliance, and safety was found to be weak or inconsistent [29]. A review of 36 primary care generic diagnostic and triage symptom checkers on web-based or mobile platforms found that appropriate triage advice was given for only 49% of the 688 vignette cases, with appropriate triage advice being given most frequently for emergency (63%) or urgent care (56%) and nonurgent care being accurate in only 30% of cases [39]. This finding is consistent with previous literature that evaluated web-based generic symptom checkers for self-diagnosis and triage [40]. A systematic review of generic health digital and web-based symptom checkers found algorithm-based triage to be inferior and more risk averse in providing appropriate triage advice compared with doctors and health practitioners [29]. Although most system developers consider this to be a safe approach, incorrect or unnecessary clinical escalations have been shown to adversely affect user trust and system adoption [41]. It is proposed that the digital assessment routing tool (DART) may overcome the limitations of existing generic symptom checkers and triage platforms by narrowing the scope of the tool and refining clinical algorithms to specifically address MSD presentations. The potential to improve triage efficiency and timely signposting of patients with MSDs to an appropriate level of care is potentially significant.

### DART Overview

**Figure 1:**
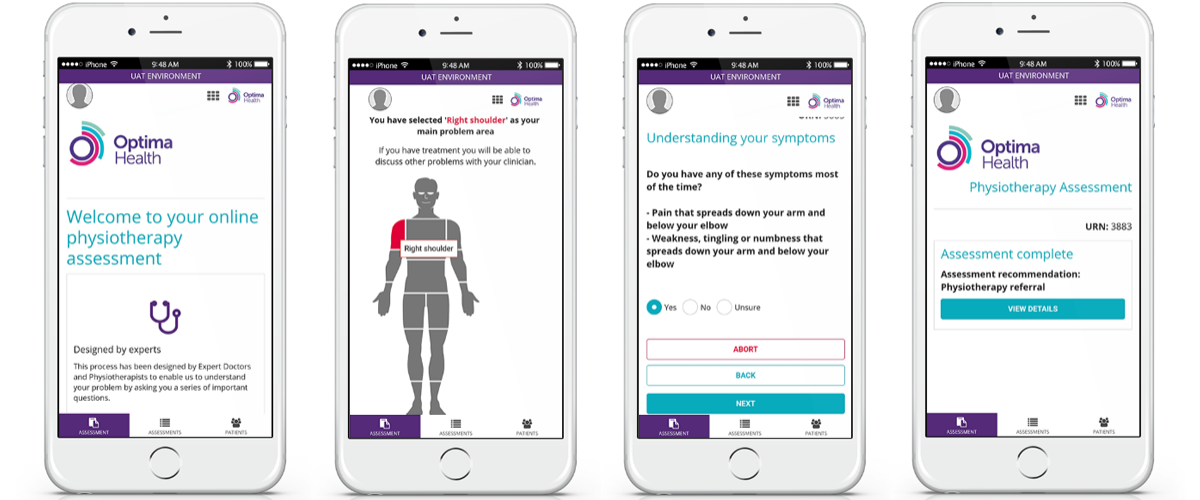
Digital assessment routing tool mobile health system.

DART (Optima Health) is the first contact mHealth system designed specifically for the management of MSDs. The clinical algorithms are configured to provide the patient with a recommendation for the correct intervention level. The patient can self-assess using a computer, tablet, or smartphone. Alternatively, the content can be delivered by a remotely situated clinician or nonclinical administrator by telephone or video call. Once the affected body region has been selected, the patient is presented with a varying number of questions, depending on the nature of their symptoms and previous responses. Serious pathology is screened for, and appropriate signposting is given at the start of the assessment, with less urgent medical referrals being identified as the patient passes through the questioning. Algorithms are configured to match the provider’s clinical services based on evidence-based practice and sector-specific referral criteria. DART can be applied across any number of health care systems, including public and private services, and typically signposts to emergency or routine medical assessment, specific condition specialists, physiotherapy, self-management programs, or psychological support services. DART has an integral reporting function that allows the analysis of individual and amalgamated patient data to assess the system and clinical pathway performance (Figure 1).

### Previous Work

This pilot study is part of a larger project, bringing DART from concept to implementation through a series of clinical and academic research work packages.

To assess the algorithm’s clinical validity, 2 reports were commissioned by Optima Health and undertaken by a panel of 5 consultant clinicians experienced in the musculoskeletal field, which comprised a consultant rheumatologist, a consultant orthopedic surgeon, a consultant sports and exercise physician and senior clinical lecturer, an honorary general practitioner (GP) in emergency care, and a consultant physiotherapist and academic lead. The first round of desktop evaluation comprised the panel inputting symptoms from 100 clinical scenarios (including red flags and complex presentations) into DART. The DART recommendation was then assessed by the panel as being correct, arguably correct, or disagree. Feedback from the panel was incorporated into a new iteration, leading to improved DART accuracy during the second review. On the basis of their opinion, the panel recommended that clinical validity was sufficient to allow DART to proceed to further research studies.

Real-world usability testing has been completed using an iterative convergent mixed methods design incorporating patient and public involvement [42], the results of which will be reported later in 2021. This study optimized usability in the final DART iteration that will be used in this pilot study and subsequent main trial.

### Research Aims and Objectives

The aim of this study is to facilitate the delivery of a future trial by exploring key aspects of trial methodology, assessing the procedures, and collecting exploratory data to inform the design of a definitive, randomized, crossover, noninferiority trial to assess DART safety and effectiveness in an NHS primary care setting.

#### Primary Objective

The primary outcome measure is to collect exploratory data about the agreement of triage decisions made by the DART system and physiotherapy-led triage, which will provide a variance (SD) estimate required for the sample size calculation in the main trial.

#### Secondary Objectives

The secondary outcome measures are as follows:

1. Evaluate the number of people who sign up and are retained, with a dropout rate and identification of when dropouts happen
2. Evaluate the systems for randomization and data collection (effectiveness, process of implementation, allocation concealment, and bias)
3. Identify the burden on the patient and therapist (treatment delay, DART procedure complexity, and additional questions)
4. Identify barriers in the proposed intervention delivery processes

## Methods

### Design

**Figure 2:**
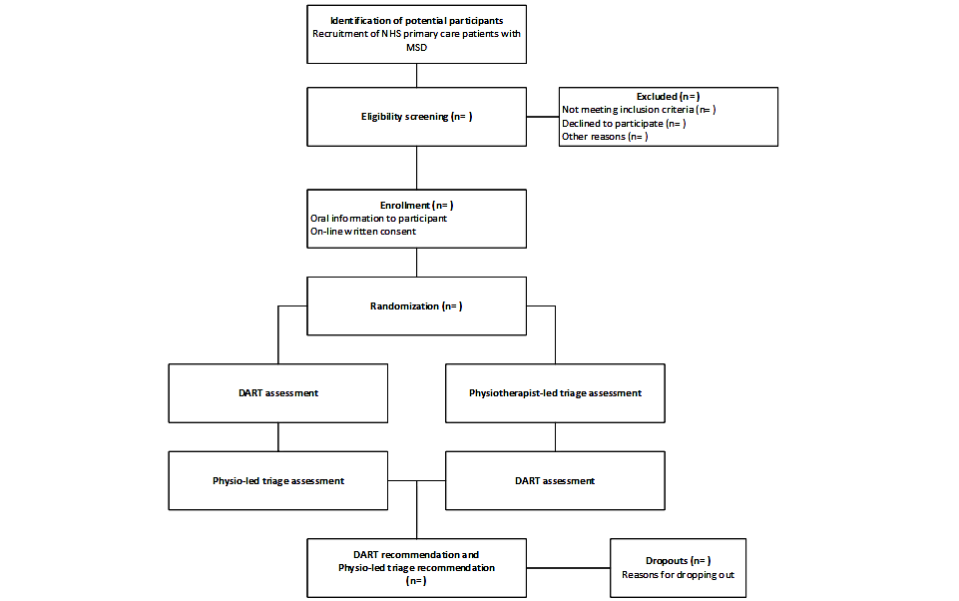
Study design and participant flowchart. DART: digital assessment routing tool; MSD: musculoskeletal disorder; NHS: National Health Service.

A pilot randomized, single-blinded, crossover, noninferiority trial will be conducted to compare the safety and efficacy of DART signposting with physiotherapy-led triage outcomes in an NHS primary care setting. This preliminary study was designed in accordance with the CONSORT guidelines for pilot and feasibility trials [43], CONSORT guidelines for equivalence and noninferiority randomized trials [44], and the CONSORT-EHEALTH (Consolidated Standards of Reporting Trials of Electronic and Mobile Health Applications and Online Telehealth) checklist [45]. Moreover, the trial design will not influence the care or triage decisions made by the usual care clinicians. Participants will complete both a DART assessment and a physiotherapy-led triage assessment on the same day, with randomization determining the order in which this is done (Figure 2).

The outcomes available to the physiotherapist will be matched by those available within the DART, allowing direct comparison between the 2 assessment outcomes (Table 1). Following their DART assessment, the participant will use a web-based questionnaire to complete the system usability scale (SUS) [46] to measure user satisfaction.

**Table 1:** All possible outcomes and suboutcomes from the digital assessment routing tool and physiotherapist-led triage assessment.

Medical care	FCP^a^ physiotherapist	Physiotherapy care	Remote self-management
Emergency care (accident and emergency referral/NHS111)	N/A^b^	Post fracture or surgery physiotherapy	Supported self-management
Urgent primary care physician (GP^c^)	Urgent FCP	Urgent physiotherapy referral	Continue self-management advice
Routine primary care physician (GP)	Routine FCP	Routine physiotherapy referral	Web-based support material
Consultant review	N/A	Physiotherapy referral plus psychosocial support	Digital self-management

^a^FCP: first contact practitioner.

^b^N/A: not applicable.

^c^GP: general practitioner.

### Recruitment

A total of 76 participants will be recruited for this pilot study using purposive sampling from a single NHS general practice. Monthly MSD referral volumes far exceed the number required for the study, which should support the completion of data collection within the anticipated study timescale. Patients with an MSD wishing to access support from the practice, either from a GP or physiotherapist, will be recruited into the study using an advert on the practice website, posters and leaflets in the GP waiting room, and by invitation from the practice administrators if the patient telephones in to make an appointment. Interested patients will be able to access the participant information sheet on the web or through hard copies available in practice, which will provide the researcher’s contact details and a request to contact them if they wish to participate (Multimedia Appendix 1). The researcher will make contact, answer any further questions, complete eligibility screening, and gain initial verbal consent. An appointment will then be made for the next available study clinic.

### Inclusion Criteria

The study participant inclusion criteria were as follows: (1) aged >18 years, (2) able to speak and read English, (3) registered as a patient at the primary care practice, (4) having a current musculoskeletal condition for which they are seeking treatment, and (5) able to access the internet either by themselves or with the help of a family or friend.

### Exclusion Criteria

The study participant exclusion criteria were as follows: (1) cognitive impairments or learning disabilities that limit a participant’s ability to follow study-related procedures, (2) unwillingness or inability to follow protocol-related procedures, and (3) Optima Health or Queen Mary University of London employees.

### Informed Consent

Each participant will receive the participant information sheet (Multimedia Appendix 1), which outlines the purpose of the study and the nature of their participation. This includes information about the format of the interaction, potential risks, confidentiality and protection of their personal data, the anonymity of study findings, and their right to withdraw at any time without prejudice. The participant will be given the opportunity to raise any questions with the researcher during the formal consenting process when they attend the practice. Formal written consent will be completed by the researcher with the patient in the practice waiting room using a web-based form. Failure to provide consent will result in the participant receiving a usual care physiotherapy-led triage without a DART assessment.

### Randomization and Blinding

Participants will be randomized to either (1) a DART assessment followed by a physiotherapist-led triage assessment or (2) a physiotherapist-led triage assessment followed by a DART assessment. This is to account for the order effects in the crossover design (Figure 2). This will be achieved by block randomization with permuted blocks of random size and without stratification factors to avoid selection bias and unequal arms [47-49]. After gaining consent, the researcher will open a sealed envelope that contains the randomization to be used for the participant. Allocation will be performed using a computer-generated list of random numbers, which will lead to a randomization sequence in Microsoft Excel 2020. The allocation ratio between the arms will be 1:1.

Blinding in this study will be ensured at 3 different points: (1) the physiotherapist leading the triage will be blinded to group allocation and DART assessment outcomes; (2) participants will be blinded to the DART assessment outcome and the physiotherapist triage outcome until they have completed both assessments and the SUS; and (3) the analysis and interpretation of the study results will be conducted by researchers blinded to the intervention group allocation.

To minimize potential bias created during the physiotherapist assessment, the physiotherapist will be required to minimize any information or advice they give to the participant until after they have completed their subjective assessment and arrived at a provisional management recommendation. This will include discussing any possible diagnoses or giving any condition management advice. Once the participant has completed both assessments and data collection is complete, they will return to the physiotherapist, who will then complete the objective assessment and continue with normal care.

### Data Collection

#### Physiotherapy-Led Triage

Participants will receive a usual care physiotherapy-led triage assessment from a first contact practitioner (FCP). An FCP physiotherapist is a qualified autonomous clinician who can assess, diagnose, treat, and discharge a patient without a medical referral, where appropriate. They have completed additional postgraduate training to provide expert assessment, check for red flags, and provide advice on self-management. If needed, they prescribe medication, order investigations, refer for physiotherapy, or provide onward referral to secondary care services such as rheumatology or orthopedics [50]. As such, the FCP physiotherapist will provide a rigorous comparator against which to measure DART signposting recommendations.

The physiotherapist-led triage assessment will be completed within a 20-minute appointment. The management pathways available to the FCPs are matched by those that can be generated by DART to allow an objective comparison (Table 1). Participants may seek help elsewhere or opt out of the study at any point, which will not affect their usual physiotherapy-led care.

#### DART Assessment

Participants will access DART using a tablet device in a treatment room or a quiet area in the practice waiting room. The researcher will log onto DART and enter the participant study number but will have no further contact with the participant until they have completed their DART assessment. The DART assessment will be completed either before or after their appointment with the physiotherapist, depending on their randomization allocation. A unique reference ID will be generated in the DART system and linked to the participant’s study number. The participant will complete the DART assessment, which will result in a signposting outcome. This will not be visible to the participant but will be stored in the DART system for later retrieval and analysis. Thereafter, the participant will complete a web-based version of the SUS, capturing their experience of using DART. All participants will be given the physiotherapist triage assessment outcome by the physiotherapist, who will complete any associated management actions or referrals. Both assessments will be completed on the same day in close succession to reduce variations in clinical presentation.

#### Panel Assessment

An independent panel comprising 3 experts in musculoskeletal physiotherapy and general practice qualified to the consultant level will provide consensus on all disagreements between DART and physiotherapy-led triage that would yield a safety concern (Textbox 1). In addition, a random sample of cases will be assessed by the panel to decide which they consider to be the correct outcome based on the patient’s presentation from the physiotherapist’s clinical record of their assessment. The panel decision will provide the definitive *gold standard* outcome against which the physiotherapist outcome will be compared. The triage outcomes that are amended by the panel will be deemed the most appropriate outcome in preference to those from the physiotherapy assessment and will provide the outcome against which DART is compared.

**Adverse triage outcomes in medical care, physiotherapy care, and self-management that would yield a safety concern, including a delay in intervention likely to result in a poor outcome.**

**Adverse triage outcomes that would yield a safety concern**

- Physiotherapy or self-management when it should have been urgent medical care (accident and emergency/NHS111 referral or urgent general practitioner)
- Self-management when it should have been physiotherapy, first contact practitioner, or medical care
- Routine care when it should have been urgent care

#### Clinical Signposting Outcomes

The primary outcome measure will be assessment outcomes from physiotherapy-led triage and DART assessments (Table 1). These decisions are classified into 4 categories (medical care, FCP, physiotherapy care, and remote self-management), with further suboutcomes in each category. This allows for precise comparisons within each category and subcategory and is based on usual care signposting approaches in musculoskeletal clinical practice. Adverse triage outcomes that could yield safety concerns will also be identified (Textbox 1). Our aim for the pilot data is to explore the agreement rate between DART and physiotherapy-led triage across assessment outcomes, with additional analysis of suboutcomes. This will inform the suitability of the outcome measure and analysis for the future main trial. Additional data will be collected from DART and the physiotherapist. Demographic variables include age and gender. Clinical characteristics include the musculoskeletal pathway related to the body site.

#### Process Outcomes

The process outcomes will help to determine whether the implementation of the main trial design is feasible. Anonymized data will be collected, including the proportion of participants who showed interest in participating against those who were recruited into the study. Participant dropout rates at each stage of the trial (and, where possible, reasons for dropping out) will be collected. System process outcomes include errors reported in randomization, allocation concealment, blinding, or data collection. Any evidence for selection bias or other sources of bias will be explored. Other outcomes include the overall time burden: average times from initial participant contact to the first assessment, along with any treatment delay because of the additional time required to perform the research procedures. Technical problems with DART or other comments from the physiotherapist or participants that pose a barrier to intervention delivery or trial procedures will be explored.

### Study Duration

Following ethics approval, 3 months will be allocated for the collection of data from the required 76 participants. The exact duration will be dependent on the volume of physiotherapy referrals entering the physiotherapy service and recruitment uptake.

### Data Analysis

#### Calculation of Main Study Sample Size

As the nature of this pilot trial is to explore trial design and feasibility, a formal power-based sample size calculation will not be conducted. Our sample size is based on the estimated stepped rules of thumb from Whitehead et al [51] to demonstrate an extra small standardized effect size (σ<0.1) at a 90% powered main trial. The obtained variance estimate of the outcome measures from the pilot data will allow sample size calculation for the main trial using the noncentral T-distribution approach from Julious and Owen [52]. Our plan is to recruit a total of 76 participants over a 3-month period in this trial.

#### Primary Outcome

The primary analysis will involve an absolute agreement intraclass correlation (A,1) estimate with 95% CIs between DART and the clinician on triage outcomes with recommended management pathways, which will be calculated using SPSS, version 23 (IBM Corporation) and based on a single rating, 2-way mixed-effects model [53,54]. The analysis includes intention-to-treat and per-protocol analyses, with a subanalysis of categories (medical care, physiotherapy care, and self-management) and adverse triage outcomes. A predefined margin of correlation with an intraclass correlation ≥0.90 will be set and based on both clinical recommendations and the literature in which a correlation was demonstrated in clinical management decisions taken between telehealth and face-to-face physiotherapy [55]. In addition, diagnostic properties (sensitivity, specificity, and predictive values) will be calculated for DART and reported with 95% CI [56]. A descriptive summary of the variables includes the mean, SD or CI, median, and IQR as appropriate. The amalgamated SUS score will be reported as a mean and used to calculate a percentile score to allow benchmarking of the DART system usability against other systems [57].

#### Evaluation of Participant Recruitment and Retention

The number of participants referring to physiotherapy will be reported, including the proportion that meets the eligibility criteria, shows initial interest, and consents to participate in the trial. Participants who show initial interest in participating but do not consent will also be reported (and, where possible, reasons for not participating). Participants who opt out of the trial at any stage, such as between interventions, will also be reported. Differences in dropout rates between the intervention groups will be compared. As recruitment rates vary in randomized controlled trials [58], a conservative margin will be set. Dropouts seem unlikely to occur as there is only a single visit that will coincide with the physiotherapy appointment. Thus, a predefined criterion of 50% and 95% will be considered satisfactory for the proportion of identified participants recruited and retained, respectively.

#### Evaluation of Randomization, Allocation Concealment, Blinding, Data Collection, and Bias

Any underpinning errors in systems responsible for procedural randomization, allocation, blinding, or data collection will be reported. Baseline characteristics will be compared between the intervention groups (DART-physiotherapy-triage and physiotherapy-triage-DART) using analysis of variance for continuous variables or chi-square tests for categorical variables. Homogeneity between groups will indicate successful randomization and minimized risk of selection bias. Discrepancies in allocation concealment or unblinding of participants or therapists will be further compared between intervention groups and reported. Unsuccessful blinding is considered when physiotherapists become unblinded to group allocation or DART assessment advice.

#### Identification of Trial Burden and Barriers to Intervention Delivery

Administrative and physiotherapist burden in terms of additional time required to administer the study process will be assessed and extrapolated to understand the implications for the main study. Feedback from the service administrators and participating physiotherapists will also be recorded and reported.

### Bias

This study is funded by Optima Health, the developers of DART, and therefore, is at risk of bias. The lead researcher (CL) is an employee of Optima Health and enrolled in a PhD program at the Queen Mary University of London. To mitigate bias, participants will be excluded if they are employees of Optima Health or the Queen Mary University of London. Participants will not have previously seen or used DART. Recruitment is through promotion directly to the medical center patients and not by the researcher contacting them using a database. There is no financial reward offered to people to participate in the study. After gaining of formal consent and ensuring that the participant has logged on to DART, the researcher has no further contact with participants, with data being collected through DART, by the physiotherapist, or the SUS web-based questionnaire. The expert panel will comprise senior musculoskeletal clinicians who are not employed in any form by Optima Health.

### Risks and Benefits

There will be no form of physical intervention during this study, and participants will have no extra travel in addition to that required for their physiotherapy appointment. Normal care, as determined by the triage physiotherapist, will be followed in all cases, and participants will have full access to all existing clinical pathways available to them. Participants will not be given the DART signposting recommendation, so there will be no conflict with the recommendation given by the physiotherapist.

### Data Management

Participants will have the right to withdraw from the study at any time. If they do, data collected up to the point they withdraw will be retained but not added to. Electronic and paper data will be managed and stored securely in accordance with the general data protection regulations. Study data will be collected outside the NHS firewall. Personal data collected in DART will be confined to age and sex at birth and linked to other research data using a DART system unique reference number. The data inputted by the participant during the DART assessment will be generated entirely by them, and no data will be extracted from their NHS records. DART system information security and data protection will be covered by Optima Health’s certification and compliance with Cyber Essentials Plus and ISO 27001. Data collected by the physiotherapist will comprise physiotherapist assessment recommendations and study numbers only. The web-based SUS questionnaire will only contain the participant’s study number. Research data will be stored separately to personal data and linked by a unique reference number that is only accessible to the researchers. Access to the participant’s physiotherapy assessment record forms a part of the study consenting process; however, only the assessment completed as a part of the study will be reviewed by the panel and no other part of the NHS record. The data collected during this pilot may be reused for a later definite randomized noninferiority trial in a deidentified format.

## Results

Ethics approval was submitted in June 2021. This study has been registered at ClinicalTrial.gov NCT04904029. Recruitment will commence early 2022, and data collection is anticipated to last for up to 3 months. The results will be reported in a follow-up paper in 2022.

## Discussion

### Overview

The demand for an mHealth system to correctly assess and signpost patients with MSDs has been demonstrated. Research into the patterned use of generic symptom checkers indicates that MSDs are among the most common reasons for accessing web-based or digital triage applications in primary care [59,60]. Studies regarding the effectiveness of generic symptom checkers have shown variable levels of system validity [23,24,29,40], and it cannot be assumed that these research findings translate to an mHealth system with a well-defined scope, such as DART with MSDs. To date, there are no published studies providing a proven methodology for evaluating the real-world validity of similar musculoskeletal mHealth systems. This pilot trial will explore the feasibility and study design for a future large-scale, noninferiority trial determining whether DART’s efficacy and safety are noninferior or *not unacceptably worse* to FCP physiotherapist-led triage, a usual practice comparator. During earlier DART development, the desktop validity of DART clinical algorithms was appraised as being good by an expert panel using vignettes; however, this is not representative of the real-world validation required for safe and effective implementation into routine clinical practice [61]. DART has demonstrated good usability through an iterative convergent mixed methods study, leading to the version to be used in this pilot trial. This pilot study protocol will give a greater understanding of how to assess the validity of an mHealth system such as DART within an NHS setting and provide a template for other researchers and developers to use across triage and referral mHealth systems.

### Methodological Limitations

The purpose of this pilot is to validate or provide information for improvement in the study design to underpin the successful completion of the subsequent main trial. The crossover design can create a delay in the participant receiving normal care, albeit only a few minutes, and there is an increase in the amount of service administration time required. The most common errors in crossover trials are failure to adapt stratification in the order of treatments and analysis of the group rather than separately between sequence groups [62]. This is accounted for by the permuted block randomization and the independent analysis of results per group. A washout period between interventions is normally recommended to prevent carryover effects; however, there is no therapeutic effect from either DART or the physiotherapy-led assessment. The physiotherapist being asked to refrain from giving management advice as they assess the patient may be a change in normal practice for some clinicians but should not affect their signposting recommendation. Carryover effects may involve the participant becoming primed to answer questions about their health differently after gaining more insight into their health problem from having completed the previous assessment. Data analysis comprises assessing clinical agreement between the physiotherapist and DART system management recommendations. This study is not designed to examine the impact of differing DART recommendations on individual patient management and how this may positively or negatively affect care complexity, case duration, or cost. This would form the basis of a future implementation study to assess these factors across the entire MSD pathway.

### Methodological Strengths

The findings from this pilot trial will constitute the design of a future noninferiority trial, as well as provide preliminary data on DART safety and effectiveness. The key benefit of a crossover design using the same participant for both arms is that differences in clinical presentation as a confounder are minimized. The large variety of MSD symptomology, patients’ general health, and psychosocial status means that using a study design where different participant results are compared, such as in a parallel study, would require an unacceptably large sample size. Assessing the participant twice within minutes reduces the risk of a change in symptoms that could lead to a different signposting recommendation between the physiotherapist and DART assessments. The use of real-world NHS participants rather than clinician testing using vignettes supports patient and public involvement, a crucial component of testing for any system designed to be used with patients. Piloting within the NHS ensures that DART is tested across a wide range of clinical presentations and patient demographics, including varying socioeconomic status, eHealth literacy, fluency of English speaking, age, and employment status.

## Appendix

Multimedia Appendix 1 Patient information sheet.

## Abbreviations

CONSORT-EHEALTH: Consolidated Standards of Reporting Trials of Electronic and Mobile Health Applications and Online Telehealth
DART: digital assessment routing tool
FCP: first contact practitioner
GP: general practitioner
mHealth: mobile health
MSD: musculoskeletal disorder
NHS: National Health Service
SUS: system usability scale
