# Supplementary material for: Validation of a Musculoskeletal Digital Assessment Routing Tool: Protocol for a Pilot Randomized Crossover Noninferiority Trial
Source: JMIR Res Protoc. 2021 Dec 13;10(12):e31541. doi: 10.2196/31541 (PMC8713101; doi:10.2196/31541)
Supplement: Multimedia Appendix 1 [file resprot_v10i12e31541_app1.docx]

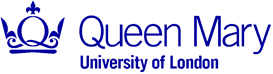


Participant Information Sheet

**Pilot study title:** Validation of a Musculoskeletal Digital Assessment Routing Tool (DART): A Pilot Randomised Crossover Non-Inferiority Trial.

**Running title:**

Does an online digital assessment tool called DART signpost patients as well as a triage physiotherapist for musculoskeletal problems?

**Researcher’s name**

Ms. Cabella Lowe

PhD student

Centre for Sport and Exercise Medicine

Mile End Hospital, London E1 4DG

c.lowe@qmul.ac.uk

**Research Ethics Committee reference number:** TBC

**Invitation**

We would like to invite you to be part of this research project. You should only agree to take part if you wish to do so; it is entirely up to you. If you choose to not take part, there won’t be any disadvantages for you, and you will hear no more about it.

Please take time to read the following information carefully before you decide whether or not to take part. This will tell you why the research is being done and what you will be asked to do should you agree to take part. Please ask if there is anything that is not clear or if you would like more information.

**What is the purpose of the study and what would taking part involve?**

One in five people in the United Kingdom suffers from a problem with their joints, muscles or other soft tissues. For these people, getting help quickly means getting better faster. Some treatments like physiotherapy or a self-help programme can help, but it can take a while to get to the right person who can help you. Some people need to see their GP first or may be asked to see or speak to a physiotherapist before they can get the right help.

Our study will explore a new way for people to quickly get the care they need using a system called DART (Digital Assessment Routing Tool). DART is an on-line system you can use on a mobile device or home computer. DART asks a series of questions and will be able to sign-post you to the right type of help for your problem. DART has been designed by a company called Optima Health, who are working with Queen Mary University of London to test the safety and effectiveness of the system.

If you wish to participate in the study, we will ask you to complete an online assessment of your problem on a tablet device in the GP waiting room, either before or after you have your appointment with the physio for your musculoskeletal problem. You will also be asked to complete a short on-line questionnaire. You will still see the physiotherapist and have any treatment you need, regardless of whether you take part in the study or not.

This is the process if you take part:

You come to the GP surgery 15 minutes earlier than your appointment . The researcher will meet you, answer your questions and get your formal consent to participate

Complete DART assessment and questionnaire on the tablet device

(15 mins)

Complete assessment with the physio

(20 mins)

The physio recommends what treatment you should have

**Why am I being invited?**

You are being invited to participate in our research study because you are an adult aged 18 years or above who shows signs of a musculoskeletal problem (a problem with the joint, muscle or soft tissue).

**Do I have to take part?**

No, it is up to you to decide whether to take part. If you do wish to consider taking part, you should read this information sheet (which you can keep) and be asked to electronically sign a consent form when you meet the researcher. You can withdraw from the study at any time without needing to provide a reason, and with no disadvantages to your usual care. Your GP will be aware that we are asking patients to participate in this study.

**What are the possible benefits of taking part?**

There is no guarantee that this study will benefit you. However, the information we gather from the trial will help us to test and improve DART, which we hope will allow people to access the right treatment quicker. There will be no changes to your treatment by taking part in this study.

**What are the possible disadvantages and risks of taking part?**

We do not anticipate any disadvantages or risks to you from taking part in this research study. The referral process will take slightly longer than usual due to the extra DART assessment. We have carefully planned the study to minimise the extra time required. You will still receive your physio assessment on the same day as you would do normally.

**Expenses and payments**

We do not offer any payment or cover expenses for your participation in this study.

**What information about me will you be collecting?**

We will be collecting the responses you have given to the questions in DART and the on-line questionnaire. The information about your musculoskeletal problem and other non-identifiable participant information, such as your age and gender, will be collected and retrieved from DART. We will also ask the physio what sort of treatment they recommended for you. In some cases, we will request the physio assessment record, which will be reviewed by a small panel of clinical experts. They will check to see how well DART agreed with the physio. In these cases, the panel will not know who you are, only your study number will be included. They will only see the record for the assessment you had for your musculoskeletal problem on that day, no other medical information will be shared.

**How will my data be stored and who will have access to it?**

Your data will be stored in a non-identifiable format. Your name and other identifiable information will be replaced by a unique code to keep your information confidential. To reduce the risk of disclosure, any personal identifiable data will be stored separately from the research data on the central servers of Optima Health and will only be accessible by the research team.

All electronic information (signed consent form, responses to questionnaire, DART assessment and treatment recommendation from the physiotherapist assessment), will be stored on the central servers of Optima Health.

**When and how will my data be destroyed?**

All your individual data collected as part of the study, will be deleted within 3 months of the study finishing.

The retention period of the de-identified study data is five years after the study has ended. This is in line with Queen Mary University of London retention schedule and information policy. The data will then be destroyed or archived.

**How will my data be used and shared?**

Your data will be stored in an anonymised format. It will be analysed, and we hope to publish the findings in a peer-reviewed medical or scientific journal.

This study is part of a larger doctoral research project developing DART and you are welcome to ask the study team for the results of the study if you wish.

**Queen Mary’s privacy notice for research participants**

Please read [Queen Mary’s privacy notice for research participants](http://www.arcs.qmul.ac.uk/media/arcs/policyzone/Privacy-Notice-for-Research-Participants.pdf) containing important information about your personal data and your rights in this respect. If you have any questions relating to data protection, please contact Queen Mary’s Data Protection Officer, Queens’ Building, Mile End Road, London, E1 4NS or [data-protection@qmul.ac.uk](mailto:data-protection@qmul.ac.uk) or 020 7882 7596.

**What will happen if I want to withdraw from this study?**

You have the right to withdraw at any time during the study without providing a reason for it. This will not have any disadvantages for you in any way. If you do withdraw, identifiable data will be destroyed securely in accordance with the UK Policy Framework for Health and Social Care research; however, data that is already anonymised or coded for analysis will not be changed.

**What should I do if I have any questions or concerns about this study?**

If you have any questions or concerns about the manner in which the study was conducted please, in the first instance, contact the researcher(s) responsible for the study. If you have a complaint which you feel you cannot discuss with the researchers then you should contact the Research Ethics Facilitators at [research-ethics@qmul.ac.uk](mailto:research-ethics@qmul.ac.uk) or Queen Mary Ethics of Research Committee, Room W104, Queens’ Building, Mile End Campus, Mile End Road, London, E1 4N or Queen Mary Ethics of Research Committee, Joint Research Management Office (JRMO) Empire House, 65 - 75 New Road, Whitechapel, London, E1 1HH. When contacting the Research Ethics Facilitators, please provide details of the name or description the study or QMERC reference number, the researcher(s) involved, and details of the complaint you wish to make.

Contact details

Researchers: Ms. Cabella Lowe

PhD student

Centre for Sport and Exercise Medicine

Mile End Hospital, London E1 4DG

[c.lowe@qmul.ac.uk](mailto:c.lowe@qmul.ac.uk)

07976 315105

Supervisor: Professor Dylan Morrissey

Professor of Sport and Musculoskeletal Physiotherapy

Centre for Sport and Exercise Medicine

Mile End Hospital, London E1 4DG

[d.morrissey@qmul.ac.uk](mailto:d.morrissey@qmul.ac.uk)

02082238839
